# Influence of Laboratory Synthesized Graphene Oxide on the Morphology and Properties of Cement Mortar

**DOI:** 10.3390/nano13010018

**Published:** 2022-12-21

**Authors:** Suganthiny Ganesh, Charitha Thambiliyagodage, S. V. T. Janaka Perera, R. K. N. D. Rajapakse

**Affiliations:** 1 Department of Civil Engineering, Faculty of Engineering, Sri Lanka Institute of Information Technology (SLIIT), Colombo 10115, Sri Lanka; 2 Faculty of Humanities and Sciences, Sri Lanka Institute of Information Technology (SLIIT), Colombo 10115, Sri Lanka; 3 Faculty of Applied Science, Simon Fraser University, Burnaby, BC V5A 0A7, Canada

**Keywords:** graphene oxide, cement, high-performance cementitious composites, calcium silicate hydrate, mechanical properties, mesopores, micropores

## Abstract

The introduction of Graphene Oxide (GO), a nanomaterial, has shown considerable promise in improving the mechanical properties of cement composites. However, the reasons for this improvement are not yet fully understood and demand further research. This study aims to understand the effect of laboratory-produced GO, using Tour’s method, on the mechanical properties and morphology of cement mortar containing GO. The GO was characterized using Fourier-transform infrared spectroscopy, X-ray Photoelectron Spectroscopy (XRD), X-ray powder diffraction, and Raman spectroscopy alongside Scanning electron microscopy (SEM). This study adopted a cement mortar with GO percentages of 0.02, 0.025, 0.03, 0.035, and 0.04 with respect to the weight of the cement. The presence of GO in cement mortar increased the density and decreased the consistency and setting times. At the optimum of 0.03% GO viscous suspension, the mechanical properties such as the 28-day compressive strength, splitting tensile strength, and flexural strength were enhanced by 41%, 83%, and 43%, respectively. In addition, Brunauer–Emmett–Teller analysis indicates an increase in surface area and volume of micropores of GO cement mortar, resulting in a decreased volume of mesopores. The improvement in properties was due to increased nucleation sites, calcium silicate hydrate (CSH) density, and a decreased volume of mesopores.

## 1. Introduction

Cementitious composites are extensively used in civil construction due to the ample availability of raw materials, well-established production processes, relatively low cost, easy usage, and high compressive strength and other properties. However, their resistance to tensile stress and crack formation remains inadequate [1]. Hence, several studies have been conducted on incorporating various natural and engineered materials into cement composites to produce high-performance composites with improved compressive and tensile strengths, durability, and crack resistance [2,3,4,5,6]. 

Within the last two decades, numerous research has been conducted on cement composites, introducing nanotechnology to modify the binder matrix and improve its performance [3,4,6,7]. The concept of changing the characteristics of cement composites at the nanoscale originates from the atomic structure of their principal hydration products, calcium silicate hydrate (CSH), and calcium hydroxide (CH). The CSH gel of Ordinary Portland Cement (OPC) has an atomic structure reminiscent of tobermorite or jennite, with multiple layers and a low degree of crystallinity at the nanoscale [8]. The CH gel exists as solid sheets when observed through SEM [7]. This offered the idea to alter the crystalline structure of CSH at the nanoscale (i.e., <100 nm) by applying nanotechnology and nanomaterials into cement composites to enhance their macro-properties.

Graphene and its derivatives, nanosheets, have caught the attention of several researchers [9,10,11,12,13,14,15,16,17,18]. Dispersion of Graphene in cement mortar is problematic and, therefore, not widely pursued [19]. Several studies have reported using Graphene Oxide (GO), a derivative of Graphene, in cement composites [6,20,21,22,23]. A monolayer of GO consists of *sp*^2^-hybridized carbon atoms and several oxygen-containing functional groups on its surface and edges. The groups include hydroxyl (–OH), carboxyl (–COOH), epoxy (–CH(O)CH–), and carbonyl (C = O) [24]. The quantity, positioning, and types of the oxide functional groups on the monolayers are random and depend on the fabrication method, chemical oxidants involved, and oxidation reaction time [25,26,27].

Numerous GO production methods have been experimented with to determine the most efficient chemical process [25,26,27,28,29,30]. Tour’s method, an improved modern version of Hummer’s method, is considered an efficient and environmentally safe GO synthesis method in the Laboratory. Improved GO produced by Tour’s method showed the presence of higher oxidation with increased *sp*^3^/*sp*^2^-hybridized carbon atoms forming the basal plane framework compared to Hummer’s method [31,32]. Hence GO preparation using Tour’s method, which involves three steps—Oxidation, Filtration, and Sonication—was adopted in this study.

Thus far, several researchers have examined the introduction of GO in cement composites [20,33,34,35]. Studies show the key to attaining superior properties by GO in cement composites is by uniform dispersion and prevention of GO agglomeration, as higher alkalinity of cement matrix leads to agglomeration [36,37]. The usage of GO suspension instead GO powder is recommended to achieve a homogeneous mix in cement composites [38]. To facilitate the dispersion of GO in the aqueous medium, mechanical methods such as Ultrasonication, high-speed mixing, and stirring are performed using minimum surfactants, such as Polycarboxylate Superplasticizer (PCE). In addition, these methods potentially break the GO layers [39,40,41]. GO in cement composites adversely affects workability by absorbing excess water due to its large surface area and hydrophilic functional groups [42]. Currently, few theories and observations state that the seeding effect of GO accelerates the hydration process and densifies the cement matrix leading to a modification in its microstructure [43,44,45,46]. The interfacial transition zone (ITZ) of the cement matrix with GO included denser hydration products on the surface of aggregates attributing to the nucleation effect of GO [47]. GO with carbon to oxygen (C:O) of almost 50:50 improved the mechanical properties of cement composites better than pristine GO of higher oxygen content because the functional groups not only act as nucleation sites but excess defects on the basal carbon frame, which reduces the mechanical properties of GO [48,49]. A review paper shows that the percentage rise of the 28-day compressive strength of cement mortar varies between 24.4% and 77.8%, cement paste varies between 13.0% and 83.0%, and concrete between 7.8% and 50.0%. Likewise, the flexural strengths of mortar and paste range from 13.7% to 70.5% and 14.2% to 90.5%, respectively [20]. GO-incorporated cement composites have better durability due to lower permeability and higher resistance to carbonation, calcium leaching, and freeze-and-thaw [50,51,52,53,54]. The findings of key past studies are summarized in Table 1.

Studies conducted using GO show its capability to elevate the properties of cement composites due to modification in its morphology. However, most of the studies reported in the literature are based on commercially available GO and their age, quality, and chemical characterization are not completely known (Table 1). A carefully controlled study that uses Laboratory produced GO powder and GO suspension of the same origin with appropriate quality control and known age together with a comprehensive chemical characterization (Fourier Transform Infrared Spectroscopy (FTIR), Raman Spectroscopy, X-ray Photoelectron Spectroscopy (XPS), XRD and SEM) of the material has not been reported with cement composites as observed in Table 1. Furthermore, important properties such as the workability and indirect tensile, compressive, and flexural strengths of the GO-enhanced cement mortar over practically relevant GO concentrations have not been reported. In addition, past studies have not confirmed the elemental composition of the hydration products shown on the SEM images through EDX (Energy Dispersive X-ray Spectroscopy) analysis. Another deficiency of existing studies is that XRD analysis has been confined only to CSH, excluding the investigation of the nature of the other hydration products such as Calcite (Ca(CO_3_)), Portlandite (Ca(OH)_2_), and others that may contribute to the role of GO in changing the properties of cementitious composites. Therefore, the objective of this study is to produce GO in the Laboratory under known conditions, obtain the chemical characterization of the GO, and conduct comprehensive chemical characterization and testing of the mechanical properties of the GO-enhanced cement mortar under controlled conditions. Through this comprehensive experimental approach, we plan to obtain a good understanding of the physical and chemical changes responsible for the enhancement of mechanical properties of cement mortar containing GO.

## 2. Methodology

### 2.1. Materials and Equipment

Sri Lanka is known for very high-quality natural graphite [61]. The natural graphite powder (99% grade carbon, ≈40 μm sized particles) was obtained from Bogala Graphite Lanka PLC, Aruggammana, Sri Lanka. All chemicals used in the GO synthesis were purchased from Sri Lankan suppliers. Deionized water (DI), with a conductivity lesser than 0.0556 μS (μ Ω−1) and resistivity greater than 18.0 MΩ.cm was used in the synthesis.

UltraTech Ordinary Portland cement (OPC) of Grade 43 adhering to ASTM C150/C150M-18 and oven-dried river sand adhering to ASTM C 144-18 and passing 4.75 mm sieve size were used in the preparation of cement mortar. Tap water used for mixing conformed to the ASTM C1602/C1602M-18 [62,63,64]. Polycarboxylic ether (PCE)-based advanced superplasticizer “Hypercrete HS” with a specific gravity of 1.07 was used in GO powder-added cement mortar.

### 2.2. Methodology

#### 2.2.1. Experimental Plan

The experiments began with the synthesis of GO in the Chemistry Laboratory, Faculty of Humanities and Sciences, SLIIT. The characterization of GO was performed at the Sri Lanka Institute of Nanotechnology (SLINTEC) Laboratory, Pitipana, Homagama, Sri Lanka, which involved FT-IR, XRD, XPS, Raman Spectroscopy, and SEM. FT-IR spectrum was acquired by Bruker Vertex 80 FT-IR spectrometer with infra-red radiation (IR) of 4000–8000 cm^−1,^ and a scanning speed of 32 cms^−1^ was used in the analysis. Raman spectra were collected using Bruker Senterra Raman Microscope with a monochromatic light wavelength of 532 nm and spectra range of 80–4500 cm^−1^. XRD patterns were collected by Bruker D8 Focus XRD instrument with X-ray wavelength of 0.154 nm, angular (2θ) range between 5° and 60° and with a step size of 0.02° (2θ). The surface chemistry was analyzed by Thermo ScientificTM ESCALAB Xi+ X-ray Photoelectron Spectrometer with a Constant Analyzer Energy of 20 eV and a step of 0.05 eV. Carl Zeiss Evo18 Research SEM simultaneously with EDAX Element Z1 analyzer EDS was used in imaging and in determining the element composition at various locations of the cement mortar specimen.

The preparation, curing, and testing of cement mortar samples were conducted at the Structural Laboratory, Faculty of Engineering, SLIIT. A few cement paste tests, including consistency and setting times, were conducted at the Cement Laboratory, UltraTech Cement Lanka. Morphological analysis tests of hardened cement mortar, including SEM with EDX, XRD, and Brunauer–Emmett–Teller (BET), were executed at the Analytical and Microscopic Laboratory, University of Moratuwa and Ceylon Graphene Technologies (CGT) Laboratory, respectively. BET analysis was acquired by Quantachrome^®^ ASiQwin TM v5.21, Autosorb iQ Station 2.

#### 2.2.2. Synthesis of GO Suspension

The improved version of Hummer’s method, Tour’s method [32], was used in GO preparation alongside mild alterations. 

For Tour’s method, 3.0 g of graphite powder was combined uniformly with KMnO_4_ of 18.0 g, with a weight ratio of 1:6, respectively. An acid mixture consisting of 360 mL of concentrated H_2_SO_4_ and 40 mL of concentrated H_3_PO_4_ (at a volume ratio of 9:1) was prepared separately. The acid mix was placed in an ice bath to control the exothermic heat it generated when added to the powder mixture. Therefore, once the acid mixture is transferred to the powder mixture, the temperature remains below 50 °C. A few minutes later, the purple solution turned dark green. The solution was left to stir at 50 °C, 550 rpm for 12 h until a purplish yellow color was observed. Then the reaction was cooled using 400 mL of deionized ice, and the excess KMnO_4_ was eliminated by treating with 3 mL of 50% concentrated H_2_O_2_.

The final solution contains impurities such as PO_4_^3−^ (from H_3_PO_4_), SO_4_^2−^ (from H_2_SO_4_), H^+^ (in acids), Mn^2+^ (from reduced MnO_4_^−^), and H_2_O_2_, in addition to the synthesized GO. The impure solution was washed with deionized water until the supernatant was free of SO_4_^2−^ ions (BaCl_2_ test) and the pH reached neutral. Finally, the impurities-free GO paste of pH 7, was dissolved in a minimum volume of deionized water and was sonicated at 60 kHz for 15 min to form the GO viscous suspension. 

The percentage of dry GO in the GO suspension was determined through the moisture content in the solution by placing the GO suspension in the oven at 80 °C for 24 h. The dried GO suspension was ground into particles and was sieved through a mesh of 2.36 mm to obtain a uniform size distribution. The GO suspension and GO powder used in the experiments below are within 2 weeks of age.

#### 2.2.3. Preparation and Testing of Cement Paste 

Cement paste was used to determine consistency and setting times. As per ASTM standard, 650 g of Ordinary Portland cement and 200 g of portal water was used to obtain the normal consistency (10 ± 1 mm) [65,66]. The overall weight of the water included was adjusted based on the water content of the GO suspension. The cement paste was mixed per the ASTM standards to obtain normal consistency [67]. Next, the cement paste was placed in a moist cabinet at 25 ± 1 °C and humidity above 95%. The initial setting time of the paste was taken once it reached 25 mm, and the final setting time was taken when the circular impression was not formed on the cement paste’s surface [65].

#### 2.2.4. Preparation and Testing of Cement Mortar Specimen

Cement mortar comprising of cement to river sand (CM/RS) ratio 1:3 and water to cement (W/CM) ratio of 0.6, with varying GO paste content w.r.t to the weight of cement, was prepared at a laboratory environmental temperature of 28 ± 2 °C. The river sand was sieved through a 4.75 mm sieve, oven dried at 110 °C for 24 h, and cooled at room temperature for 12 h before casting [63]. Potable water of 26 ± 1 °C was used to mix mortar [62]. GO suspension required different mix ratios, as represented in Table 2, and was obtained from the same batch of GO suspension. The ages of GO samples used in all mixes were within two weeks from the synthesis. Another set of cement mortar of the same CM/RS ratio was cast using dry GO powder, Polycarboxylate Superplasticizer (PCE), and a W/CM ratio of 0.4. PCE was used to ensure normal workability with a low W/CM ratio of 0.4, as with an increase in GO, the workability of cement mortar decreases. The powder was dispersed well in PCE and water using an ultrasonic bath sonicator (60 kHz) for 10 min. Cement mortar was cast into molds of 50 mm × 50 mm × 50 mm cubes, 40 mm × 40 mm × 160 mm beams, and 80 mm × 160 mm cylinders [68,69]. The cylinders for splitting tensile strength were adopted from concrete tests [70]. The casting of beams, cylinders, and cubes was performed per the ASTM standards, ensuring constant compaction energy by means of a concrete vibrating table.

Test specimens detached from the molds after 24 h were cured in saturated Ca(OH)_2_ water, maintaining a concentration of 3.0 g/L and at a temperature of 26 ± 0.5 °C [71]. The specimens were tested for compressive strength at 7 and 28 days and split tensile strength at 28 days, with a loading rate of 0.3 kN/s, using a universal testing machine (UTM). The flexural strength of each specimen was determined using a lower loading rate of 0.1 kN/s. The splitting tensile strength and flexural strength was calculated based on Equations (1) and (2), respectively [69,70]. The stated values in the results section are the averages of at least three specimens with an allowed derivation of 10%, and any values outside the range were considered an outlier.

Splitting tensile strength equation:

T = 2P/πLD,(1)

where T is the splitting tensile strength (MPa), P is the maximum load (N) applied by the UTM, L is the length of the cylinder (mm), and D is the diameter of the cylinder (mm).

Three-point flexural strength equation:

σ = 3FL/2bd^2^, (2)

where σ is the flexural strength (MPa), F is the maximum load (N) applied by the UTM, b is the width of the beam (mm), and d is the length of the beam (mm).

## 3. Results

### 3.1. Characterization of GO Produced in Laboratory

**Figure 1:**
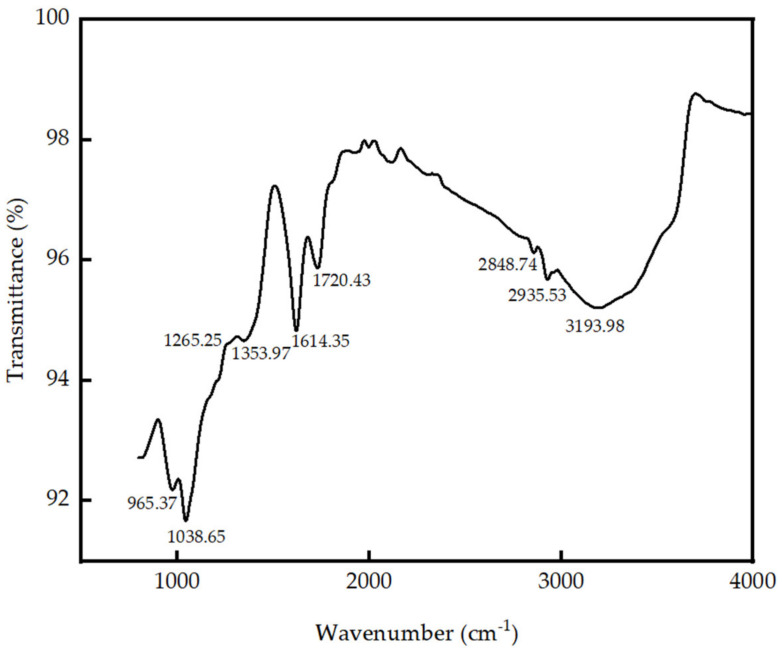
FT-IR of Graphene Oxide.

The broad peak at 3193.98 cm^−1^ of the FT-IR spectrum of GO (Figure 1) is attributed to the O–H stretching, while the peak at 1720.43 cm^−1^ is assigned to the saturated C = O group of carboxylic acids or conjugated C = O bond of esters. The presence of the C–O bond in ester/ketone/aldehyde/carboxylic acid is represented by the peaks at 1038.65 cm^−1^ and 1265.25 cm^−1^. C = O stretching vibration of an ester/ketone/aldehyde/carboxylic acid is represented by the peak at 1728 cm^−1^. Hence, it is evident that carboxylate, ester, lactone, and aldehyde functional groups are present in GO which is created by the oxidation of the carbon surface. The peaks at 2848.74 and 2935.53 cm^−1^ are attributed to the H–C stretching, and the peak at 1353.97 cm^−1^ represents the C–H bending. The conjugated C = C bonds of alkenes are represented by the peaks at 1614.35 cm^−1^ and 965.37 cm^−1^, respectively [72].

**Figure 2:**
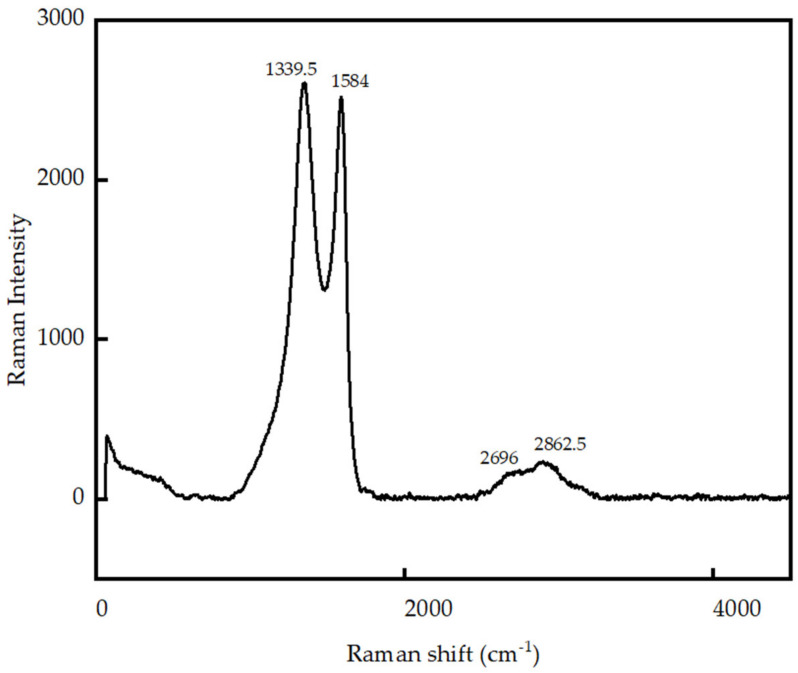
Raman spectroscopy of Graphene Oxide.

The Raman spectrum of GO, shown in Figure 2, exhibits the G and D bands appearing at 1339.5 cm^−1^ and 1584 cm^−1^, respectively, and the wide 2D band at 2696 cm^−1^. The G band is the primary mode in graphene-based material, representing bond stretching of carbon atoms in sp2 chains and rings. The existence of the D band specifies a higher degree of disorder, which arises due to gaps and defects formed in sp2 carbon rings due to oxidization heading to sp3 hybridized carbon. The 2D band originates due to the second-order Raman scattering process.

The intensity ratio (ID/IG) measures the structural disorder of carbon-based materials. The ID/IG ratio of the synthesized GO was found to be 1.034, revealing that almost equal sp3 and sp2 carbon rings are present in GO. The 2D band indicates graphene oxide layer thickness, and the wide 2D band demonstrates that the GO is stacked, increasing its thickness [73].

**Figure 3:**
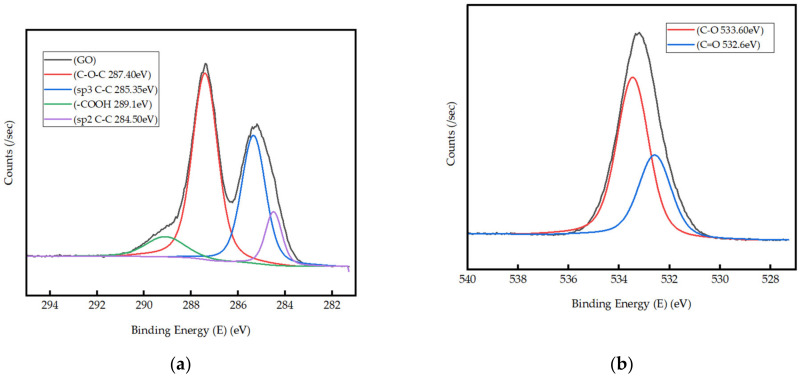
(**a**) Narrow XPS of C1s; (**b**) Narrow XPS of O1s.

XPS higher resolution spectra were acquired to study the surface chemistry of synthesized GO (Figure 3a,b). The higher resolution spectrum of C1s (Figure 3a) is deconvoluted into four peaks centered at 284.5, 285.35, 287.40, and 289.1 eV, which could be attributed to C–C sp2 hybridized carbon, C–O, O = C–O bonds and satellite feature, respectively [74]. The sub-peaks in the higher resolution spectrum of O 1s of GO (Figure 3b) appear at 532.6 and 533.6 eV and are assigned to C = O and O–C = O bonds, respectively [75]. Based on the atomic percentages of carbon and oxygen, the average carbon-to-oxygen (C/O) ratio is calculated to be 1.90. XPS results are consistent with the FT-IR analysis and further confirm the presence of functional groups such as ester/ketone/aldehyde/carboxylic acid.

### 3.2. Physical Properties of Cement Composites with GO Paste and Powder

This section covers the physical properties of GO powder- and GO paste-incorporated cement mortar alongside consistency and setting times for GO paste-added cement paste. Consistency and setting times of GO powder-added cement paste were not performed due to non-uniform readings, which could have resulted due to the inconsistent dispersion of GO powder. The wet densities reported are the fresh wet densities of cement mortar onset of casting, whereas the dry densities are the 28-day hardened cement mortar samples, oven-dried for 24 h at 105 °C. The 7-day and 28-day compressive, flexural, and splitting tensile strengths of GO powder and GO paste-enhanced cement mortar are shown below. The terms “7-day” and “28-day” define the number of days the samples have been cured under lime water prior to testing. All results stated below are a mean of at least three samples with a derivation below 10% from the mean. GO sample identified as “0% GO” represents the control cement mortar, and samples labeled as “0.03% GO” mean 0.03% of GO w.r.t to the weight of cement has been incorporated in the mortar. In addition, the ensuing figures showing the physical properties are plotted to show the maximum, minimum, and average values of the test samples at each GO percentage considered.

#### 3.2.1. Consistency and Setting Times of GO Paste Incorporated Cement Paste

**Figure 4:**
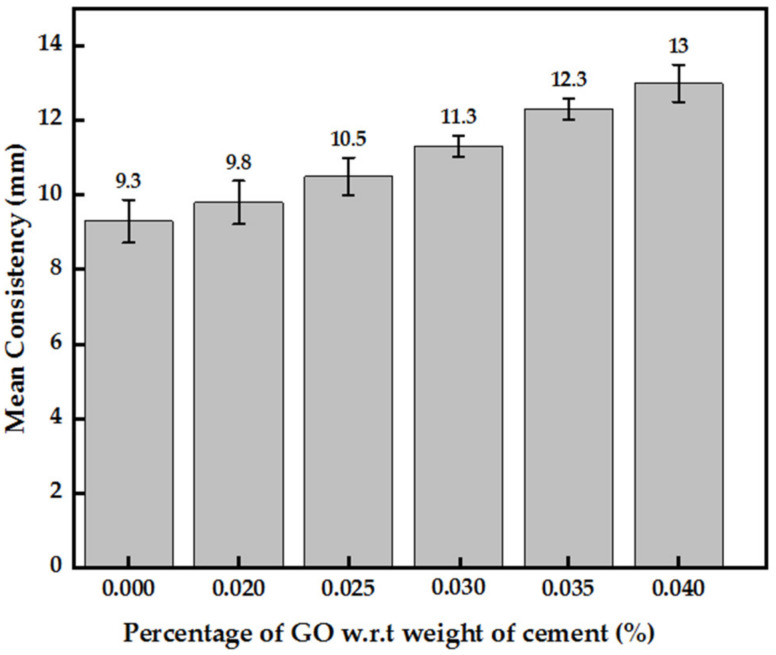
Consistency of cement paste-incorporated with GO paste.

In general, the consistency of cement paste rises with an increase in GO paste concertation, indicating a drop in workability, as shown in Figure 4. A uniform increment in consistency by about 7–10% with each 0.005% increase in GO percentage was observed. The maximum increase in consistency of roughly 40% was observed with 0.04% GO.

**Figure 5:**
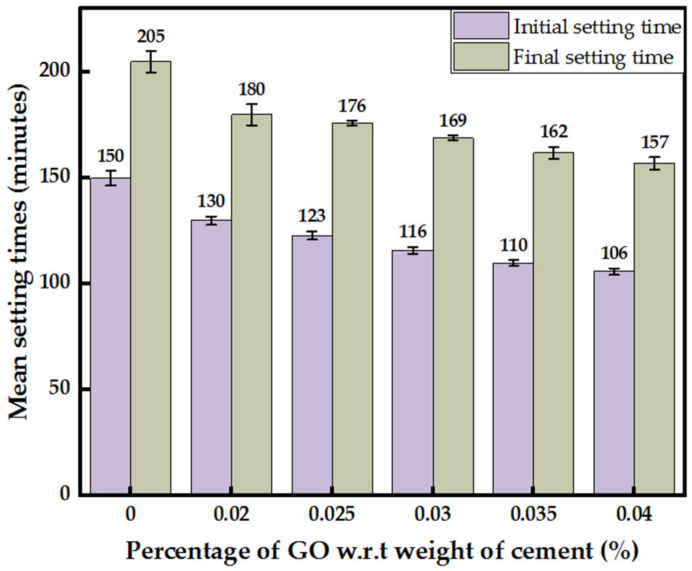
Initial and final setting times of cement paste incorporated with GO paste.

An overall decrease in initial and final setting times with an increase in GO percentage was observed, as shown in Figure 5. Maximum drop in initial and final setting times was spotted with 0.04% GO by 30% and 24%. A rise in GO percentage by 0.005% decreased the initial and final setting times by an average of 6 min. However, the difference between initial and final setting times with varying GO percentages remained constant at about 50 min for the GO percentage range considered.

#### 3.2.2. Densities of GO Paste- and GO Powder-Incorporated Cement Mortar

**Figure 6:**
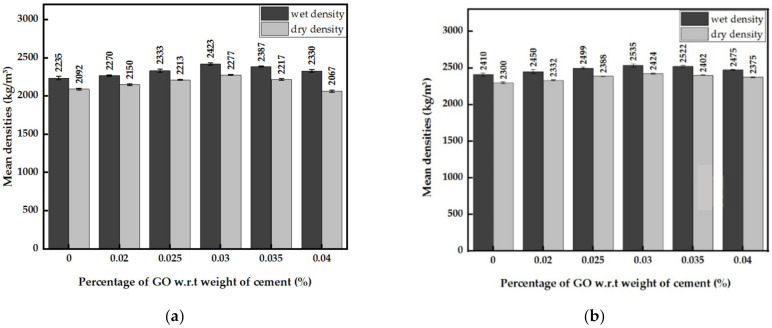
(**a**) Wet and dry densities of cement mortar with GO paste; (**b**) Wet and dry densities of cement mortar with GO powder.

The graphs in Figure 6a,b show increases in the wet and dry densities until 0.03% GO and a drop afterward. With the presence of GO, both densities show increases compared to the control cement mortar for the GO percentage range considered. At the optimum value of 0.03% GO concentration, the wet and dry densities of GO paste-containing cement paste improved by approximately 9%. In contrast, with GO powder, the wet and dry densities increased by roughly 5%. The wet and dry densities of GO powder-incorporated cement mortar were slightly larger than that of GO paste-incorporated cement mortar (W/CM of 0.6). It is due to the lower W/CM ratio (0.4) of the mortar containing GO powder and the PCE superplasticizer in the cement mortar. The peak wet and dry densities at the optimum GO percentage were 2423 kg/m^3^ and 2277 kg/m^3^ for mortar with GO paste, and 2535 kg/m^3^ and 2424 kg/m^3^ for mortar with GO powder.

#### 3.2.3. Mechanical Strengths of Mortar with GO Paste and GO Powder

**Figure 7:**
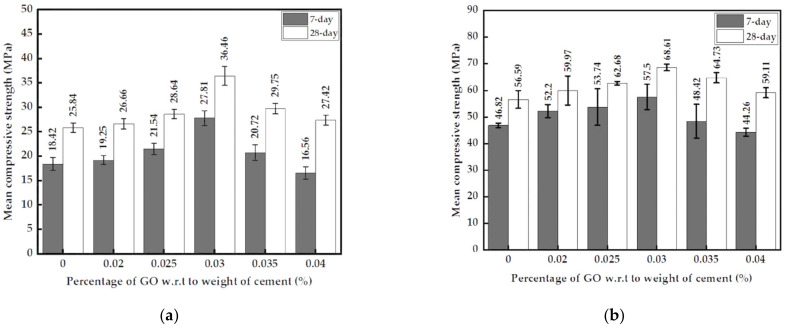

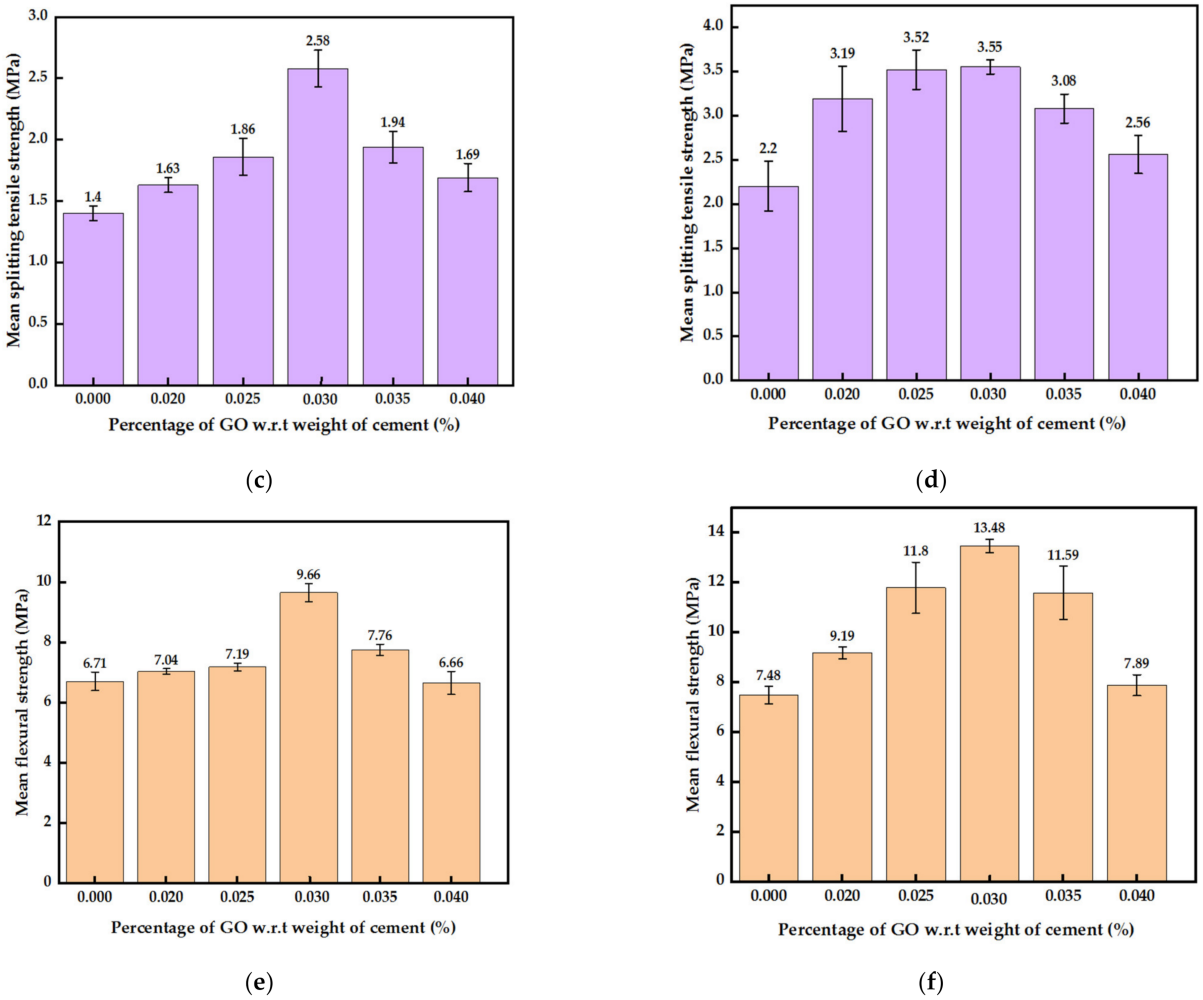
(**a**) 7-day and 28-day compressive strengths of cement mortar with GO paste; (**b**) 7-day and 28-day compressive strengths of cement mortar with GO powder; (**c**) 28-day splitting tensile strengths of cement mortar with GO paste; (**d**) 28-day splitting tensile strengths of cement mortar with GO powder; (**e**) 28-day flexural strengths of cement mortar with GO paste; (**f**) 28-day flexural strengths of cement mortar with GO powder.

The 7-day and 28-day compressive strengths increased until 0.03% GO for mortar with both paste and powder and then decreased with increasing GO percentage, as shown in Figure 7a,b. The compressive strengths of cement mortar at the optimum 0.03% GO paste (Figure 7a) showed improvements of 7-day and 28-day strengths by 51% and 41%, respectively, compared to a sample without GO. The 7-day and 28-day compressive strengths of mortar with GO powder (passing 2.36 mm sieve) showed increases of 23% and 21%, respectively, as shown in Figure 7b. It is also noted that at 0.04% and beyond, the 7-day strength was below the value for the control sample. The mortar with GO powder generally has higher strengths at 7 and 28 days compared to the same with GO paste, as the W/CM ratio of the mortar with powder is lower and contains PCE superplasticizer. However, the gain in strengths at the optimum level of GO is much better for the mortar with the paste than the mortar with the powder.

The maximum splitting tensile strength of mortar with GO paste or powder was also observed at 0.03% of GO (Figure 7c,d). In addition, the trend of tensile strength with varying GO percentages is similar to the case of compressive strength. GO in powder or paste form causes a strength enhancement within the range of GO percentages considered. The splitting tensile strength of mortar with GO paste increased by 84% at 0.03% concentration, whereas the maximum increase was 62% for mortar with GO powder. However, the peak tensile strength of mortar with GO powder is nearly 50% higher than the corresponding value for cement mortar with GO paste.

The results shown in Figure 7e,f also confirm a trend that is similar to the trends observed for compressive and splitting tensile strengths. The optimum GO percentage is 0.03% for the flexural strength of mortar with GO paste and powder. The maximum strength increase is around 44% for mortar with GO paste. However, mortar with GO powder shows a maximum strength increase of 80%. As seen previously for other strength parameters, the flexural strength of mortar with the GO powder is higher than that for mortar with GO paste due to the difference in the W/CM ratio and the presence of the PCE superplasticizer.

### 3.3. Morphology of Cement Mortar with GO Paste

#### 3.3.1. Interpretation of SEM and EDX Results

**Figure 8:**
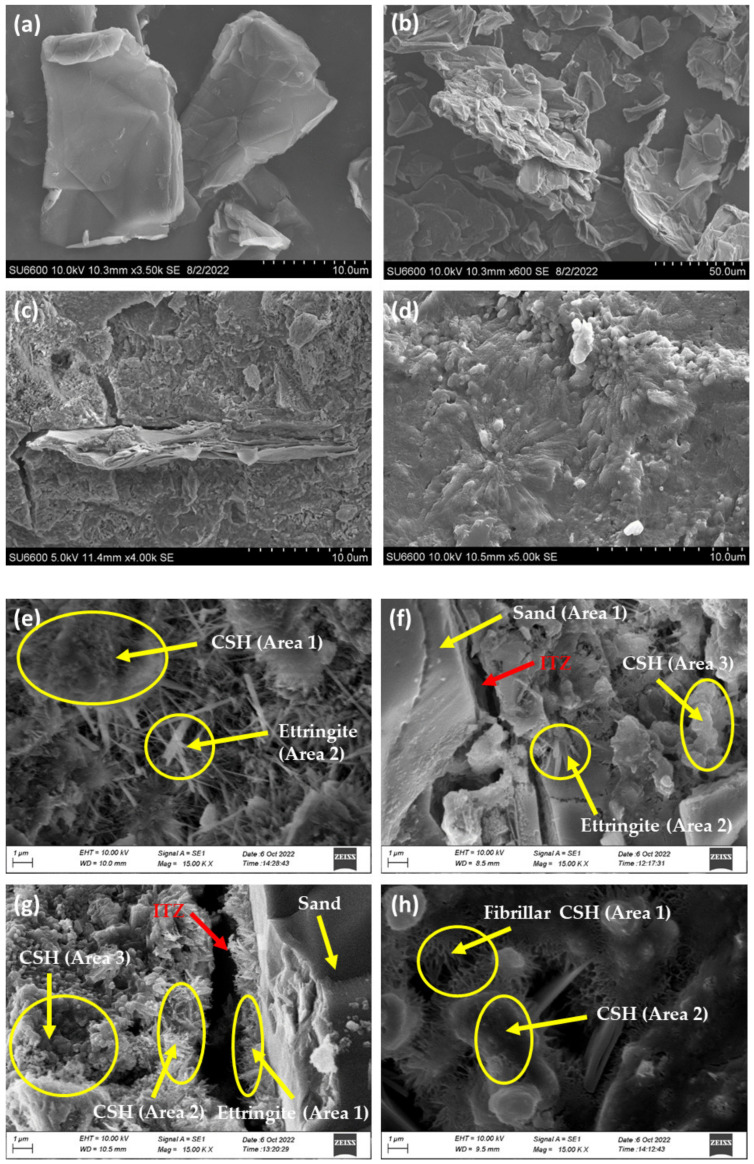
(**a**) GO sheets (at 3500 magnification); (**b**) GO sheets (at 600 magnification); (**c**) SEM of GO added mortar on a fracture surface; (**d**) SEM of GO added mortar from the top view; (**e**) SEM of control mortar; (**f**) SEM of 0.02%GO added mortar; (**g**) SEM of 0.03%GO added mortar; (**h**) SEM of 0.04%GO added mortar.

SEM images were collected to study the morphology of the GO and cement composites. SEM images of GO shown in Figure 8a,b exhibited the crumpled and wrinkled lamellae structure resulting from the oxidation of graphite. The homogeneous graphene nanosheets are folded, and the edges of the individual nanosheets, including the kinked and wrinkled areas, are well distinguishable. The wrinkles in the GO sheets are produced due to the presence of oxygenated groups. Oxidation occurs at the edges and surface of the graphite flakes. It extends further to the carbon located in the middle, creating oxygenated groups between the sheets, increasing the inter-layer distance. Ultrasonication further increases the interlayer distance during the preparation of GO suspension.

A fracture surface SEM image shown in Figure 8c exhibits a multilayered GO sheet surrounded by CSH gel. A few CSH products on the surface of GO and propagation of CSH from the GO sheet are observed on the right side of the image. Figure 8d indicates GO acts as a nucleation site to CSH formation, as a pulling effect of CSH towards the center was spotted at several sites of 0.03% GO cement mortar. However, the presence of GO particles was not observed from the top view of the image. This is due to GO particles smaller than shown in Figure 8c and the particles present beneath the imaging surface; hence only the effect was observed.

SEM images of the cement mortar prepared without GO (Figure 8e) showed disorderedly located needle and bar-like crystals. Such appearance is comparatively lower in GO-incorporated cement samples, and the cotton-like crystals are abundant (Figure 8f,g).

The SEM image of the control cement mortar (Figure 8e) shows the needle-like ettringite crystals on CSH, as indicated in the image. Similar areas are seen on the SEM images of mortar with 0.02% GO (Figure 8f). However, in the SEM image of mortar with 0.03% GO, more cotton-like CSH crystals (C_3_S) (Figure 8g) are observed. Furthermore, the Interfacial Transition Zone (ITZ) of 0.02% GO-incorporated cement mortar showed no hydration products on the surface of Sand (SiO_2_). In contrast, mortar with 0.03% GO exhibited more CSH crystals, increasing the thickness of the ITZ. Interestingly, in the SEM image of 0.04% GO incorporated cement mortar composite (Figure 8h), fibrillar CSH was formed on C_3_S areas.

EDX spectra of the cement composites were collected to study the elemental composition (Table S1). EDX spectra of the control cement mortar taken at Area (1) and Area (2) with different morphologies show different compositions. Area (1), comprised of a needle-like structure, consists of Ca with an atomic percentage of 38.24%, while Area (2), with the cotton-like morphology, consists of Ca with an atomic percentage of 19.12%. The Ca percentage at Area (1) is greater than that of Area (2), indicating that the chemical formula of needle-like morphology is Ettringite (Ca_6_Al_2_(SO_4_)3(OH)_12_·26H_2_O) and that of cotton-like morphology is CSH (Ca_2_SiO_4_·30H_2_O). EDX spectra were collected from three different areas of 0.02% GO incorporated cement mortar denoted as Area (1), (2), and (3). Area (1) corresponds to sand (SiO_2_) as the highest atomic percentage (30.64%) next to Oxygen belongs to Si compared to Ca (1.10%), and Area (2) and (3) represent Ettringite and CSH, respectively, as indicated by high Al content in Ettringite compared to CSH. Area (1), (2), and (3) of cement mortar with 0.03% GO are identified by the EDX spectra. They mainly consisted of Area (1): sand (SiO_2_) and Ettringite on the surface of the sand, Area (2): Ettringite, Area (3): CSH, and other compounds in relatively small quantities. Area (1) and Area (2) of mortar with 0.04% GO mainly consist of fibrillar CSH and CSH, respectively, as shown in the EDX spectra of those areas.

#### 3.3.2. Interpretation of XRD Results

**Figure 9:**
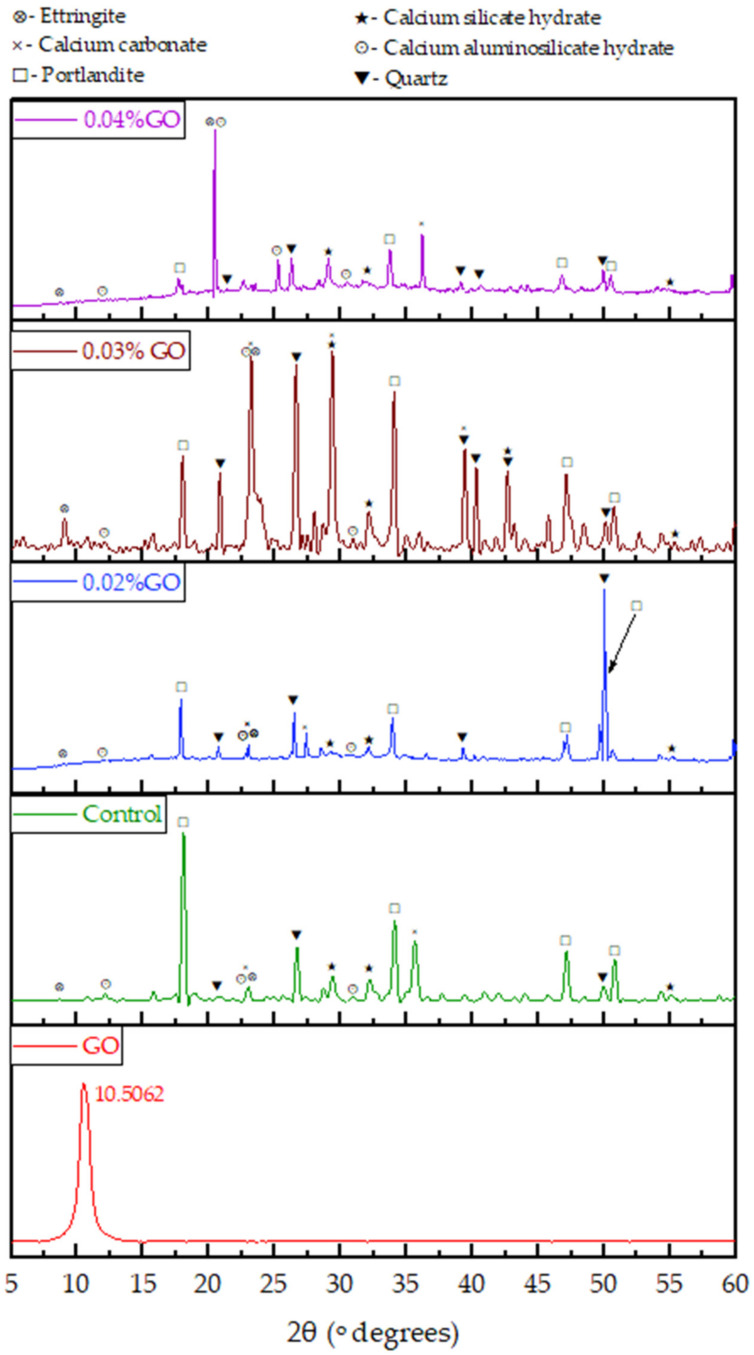
XRD analysis of GO and cement mortar with GO.

The XRD of GO, shown in Figure 9, displays a sharp peak at 10.5062° (2θ) with a full width at half maximum (FWHM) of 0.898°, indicating a higher crystallinity. The distance (d) between the adjacent GO layers was calculated as 0.841 nm, according to Bragg’s law shown in Equation (3), confirming that several GO layers are stacked together.

Bragg’s law equation:

λ = 2dsin(Ɵ),(3)

where λ is the wavelength of the bombarded X-ray beam (0.154 nm), θ is the diffraction angle (θ = 5.253°), and d is the interlayer spacing between the GO sheets.

X-ray diffraction patterns were collected to determine the crystallography of the composites (Figure 9). XRD pattern of the cement mortar shows diffraction peaks corresponding to SiO_2_, Ca(OH)_2_, Calcium aluminum silicate hydrate (CASH), Calcium silicate hydrate (CSH), CaCO_3,_ and Ettringite. The peaks appearing at 20.67°, 26.71°, and 49.96° are attributed to SiO_2_ (ICCD–00-004-0733) whereas the peaks at 18.10°, 34.07°, 47.09°, and 50.80° are assigned to Ca(OH)_2_ (ICCD–00-004-0733). The presence of CASH is confirmed by the peaks at 12.14°, 23.03°, and 30.96° (ICCD–00-046-0341), and the peaks appearing at 29.45°, 32.26°, and 55.05° reveal the presence of CSH (ICCD–00-015-0584). Further, the peaks at 23.03° and 35.63° confirm the presence of Calcite (ICCD–01-086-2339), and the peaks at 8.69° and 23.03° indicate the presence of Ettringite (ICCD–00-002-0059). The crystallite size of Ca(OH)_2_ and CSH of the control cement mortar and 0.03% GO incorporated cement mortar were calculated using the peaks at 18.10° and 29.45°, respectively.

The d-spacing of Ca(OH)_2_ of mortar with 0.03% GO is 0.49715 nm. It is greater than that of pure cement mortar (0.4901 nm). Similarly, the d-spacing of CSH of mortar with 0.03% GO is 0.2795 nm. It is greater than that of the control mortar (0.2778 nm).

#### 3.3.3. Interpretation of BET Results

**Figure 10:**
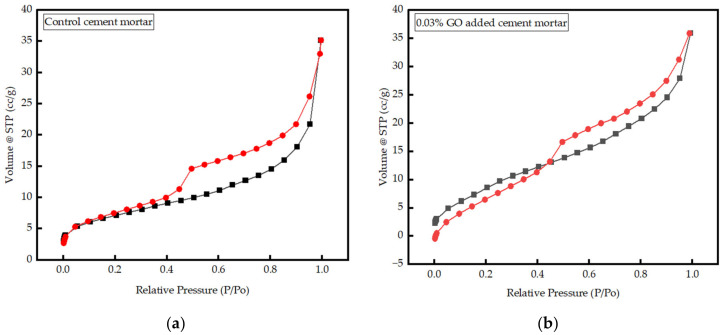
Isotherm graphs for Absorption and Desorption (**a**) control (0% GO) cement mortar; (**b**) cement mortar with 0.03% GO.

**Figure 11:**
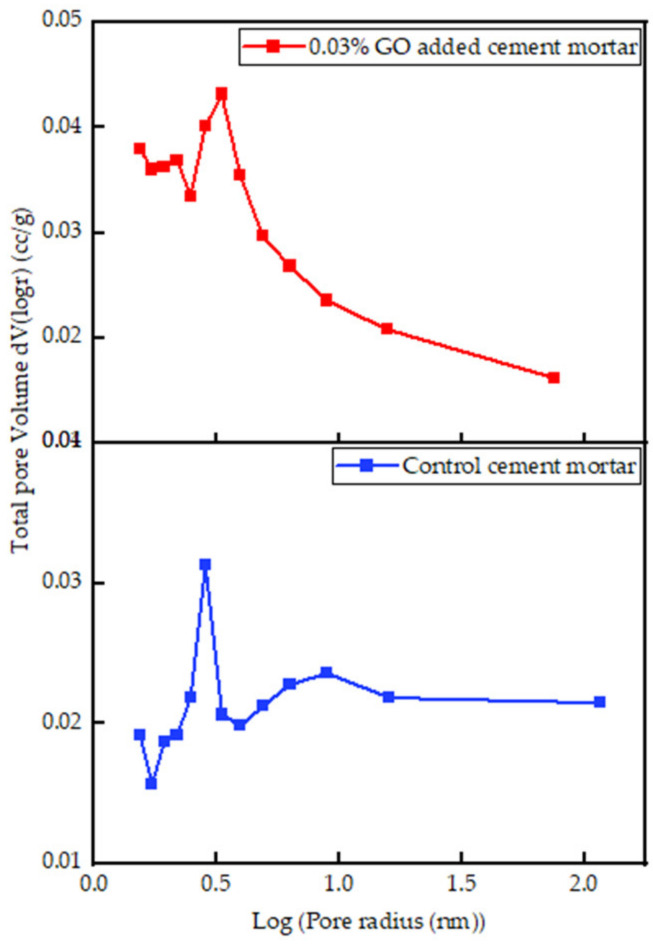
Total pore volume against pore radius of control and 0.03% GO-added mortar.

The BET analysis was performed on control cement mortar and mortar with 0.03% GO. Figure 10a,b show the adsorption and desorption isotherms, and the BJH pore size distribution curves are exhibited in Figure 11. Both compounds show type IV isotherms commonly found in mesoporous materials. The BET surface area of cement mortar (25.18 m^2^/g) is increased to 36.77 m^2^/g with the incorporation of 0.03% GO. The total pore volume and the micropore volume of the control mortar were estimated as 0.047 cc/g and 0.010 cc/g, respectively. Meanwhile, in the mortar with 0.03% GO, the total pore volume (black) remained the same (0.047 cc/g), but the micropore volume (red) slightly increased to 0.011 cc/g. In addition, the pore radius of mortar with 0.03% GO is 1.54 nm and identical to the pore radius of the control mortar. An increase in the volume of micropores and a decrease in the volume of mesopores were caused due to increased quantities of hydration products in cement mortar with 0.03% GO compared to the control mortar. More hydration products enhance the strength of the cement matrix, as more energy is required for failure.

## 4. Discussion

GO was produced in the Laboratory using Tour’s method with mild modifications, such as cooling the acid mix before mixing to avoid sudden exothermic reaction and loss of graphite from the mixture. Sonication was used to ensure a uniform solution in the cement paste or mortar without any lumps of GO. In determining the moisture content and during the production of powdered GO, the GO suspension was exposed to 80 °C. It was assumed that GO would not reduce at 80 °C as studies have shown complete reduction happens at about 550 °C [76]. The oxygen-containing functional groups observed in the FTIR and XPS of the synthesized GO confirm the presence of hydroxyl (–OH), carboxyl (–COOH), epoxy (–CH(O)CH–), and carbonyl (C = O) fitting the schematic diagram of graphene oxide (GO) in Figure 1. XRD results confirm that synthesized GO has a higher crystallinity with an FWHM of 0.898° and an interlayer spacing of 0.841 nm between the stacked GO sheets.

Addition of GO viscous suspension (<200 μm) and GO powder (<2.36 mm) into cement mortar improves its mechanical properties, especially at the optimum percentage of 0.03% GO. The 7-day and 28-day compressive strengths of mortar with 0.03% GO paste increased by 51% and 41%, respectively, and the 28-day splitting tensile and flexural strengths increased by 84% and 44%, respectively. With an optimum concentration of 0.03% GO powder, the 7-day and 28-day compressive strengths, splitting tensile strength, and flexural strength increased by 23%, 21%, 62%, and 80%, respectively. In general, the deviation of results with GO powder was larger than with GO paste, as it is difficult to attain a uniform mixing with powder. Consistency and setting times tests performed on cement pastes with GO suspension showed an increase in consistency and a decrease in initial and final setting times. However, the difference between setting times remained constant at about 53 min. In addition, the presence of GO increased wet and dry densities of mortar by roughly 5%, with the optimum values observed at 0.03% GO.

The SEM images of GO-containing cement mortar showed that GO acts as a nucleation site due to the “pulling effect” and the presence of CSH on GO sheets. The control mortar displayed higher intensity of Ettringite (Ca_3_Al(OH)_6_·12H_2_O), shown by thin rod-like crystal projections, on CSH (3CaO·2SiO_2_·4H_2_O). The cement mortar with 0.02% GO showed lesser rods and more CSH. The cement mortar with 0.03% GO showed no thin rod crystals; instead, tobermorite CSH crystals were observed. With 0.04% GO, a disrupted matrix with fibrous CSH was observed. This indicates that the presence of tobermorite CSH is a key ingredient for strength gain at 0.03% GO, and the existence of Ettringite and fibrous CSH causes a loss in strength. Furthermore, the interfacial transition zone of mortar with 0.03% GO had more CSH on the surface of the sand (SiO_2_), increasing the thickness of the zone, which improves resistance and strength. The pure CSH gel in 0.03% GO cement mortar is more crystalline than Ettringite in the control and mortar with 0.02% GO, whereas fibrous CSH was observed in the mortar with 0.04% GO.

BET analysis was used to determine the overall pore volume, micropores volume, pore radius, surface areas of control, and 0.03% GO cement mortars. For mortar with 0.03% GO, BET analysis showed an increase in the surface area by 46% while keeping the overall porosity and pore radius identical to the control mortar. The volume of micropores increased by 10%, which indicated no significant change in the overall porosity of the material. However, the volume of micropores (<100 nm) has increased, indicating a lesser amount of mesopores (0.01 mm to 1 cm) in the mortar due to the presence of GO. A decrease in the volume of mesopores resulting from an increase in the content of hydration reaction products leads to improved mechanical strength properties of cement mortar.

## 5. Conclusions

This study concludes the following:

- With each 0.005% increase in GO percentage, a uniform increment in the consistency by about 7–10% was recorded, while a decrease in the initial and final setting times by an average of 6 min with the difference between the initial and final setting times constant at about 50 min was observed.
- The optimum percentage of GO paste, at 0.03%, increased the 7-day and 28-day compressive strength, splitting tensile strength and flexural strength by 51%, 41%, 84%, and 44%, respectively. The increment in compressive and splitting tensile strength with the optimum 0.03% GO powder added mortar was lesser than GO paste-containing mortar. The substantial increase in tensile strength is a major advantage as it would increase the toughness of the cementitious composite.
- Oxygen functional groups act as nucleation sites by attracting Ca^2+^ ions to them. Hence, CSH and Ca(OH)_2_ formed on the sites have a more organized structure compared to cement mortar in the absence of GO, improving the strength properties and increasing densities.
- The volume of micropores in 0.03% GO mortar increased by 10% compared to the control, keeping the overall porosity constant, leading us to the conclusion of reduced mesopores in GO-containing mortar.

The application of GO in practical situations is still not clear as a complete understanding of the role of controlling variables, such as the GO quality and age, W/CM ratio, cement type, admixtures, and other factors are not fully understood, and the cost of GO remains a major barrier. In addition, the durability of GO-incorporated cement composites is also not well-researched. Hence, future studies on the impact of GO on the durability of cementitious composites and related morphological examinations would be valuable in further advancing the use of nanomaterials in cementitious composites. The potential of hybrids of different nanomaterials with GO is another topic that deserves further research, especially to reduce cost. Research on cheaper production methods of GO is necessary to make GO a viable material in the construction industry.

## Figures and Tables

**Table 1:** Summary of key findings of past studies on GO incorporated cement mortar/cement paste.

Reference	Source and Age of GO	Optimum Dosage of Dry GO %.	Percentage Change in Properties (%)				Summary on Morphological Study
			Workability	Compression	Tension	Flexural	
[47]	Hummer’s method, age of GO unknown.	0.03%	-	3-day increased by 45.1%, and 28-day increased by 38.9%.	(Direct method) 3-day increased by 51.0% and 28-day increased by 78.6%.	3-day increased by 70.7% and 28-day increased by 60.7%.	Flower-like hydration crystals formed via nucleation effect of GO.
[55]	Modified Hummers method, age of GO unknown.	1.5%	-	-	(Direct method) 28-day increased by 48%.	-	More calcium silicate hydrates (CSH) formed in presence of GO.
[56]	GO suspension purchased, age of GO unknown.	0.02%	Flow table test, workability reduces by 2.8%.	3-day increased by 34.4%, and 28-day increased by 25.9%.	(Splitting method) 7-day increased by 21%, and 28-day increased by 18%.	28-day increased by 14.8%.	Densification of ITZ and pore structure modification, in the presence of well dispersed GO.
[44]	GO powder purchased, and age of GO unknown	0.2%	-	28-day increased by 23.4%.	-	28-day increased by 58.9%.	A refining effect on the volume and diameter of the pores is observed. The volume of loosely packed CSH reduced while the volume of highly packed CSH increased with the addition of GO.
[45]	GO suspension purchased, and age of GO unknown.	0.03%	-	-	-	-	The increased quantity of gel pores enhanced the microstructure of cement paste, improving properties such as water sorptivity and chloride penetration.
[46]	GO suspension purchased, and age of GO unknown.	0.10%	Mini slump test, flowability reduces by 6.0%.	14-day increased by 18%, and 28-day increased by 20.5%.	-	14-day increased by 15%, and 28-day increased by 28%.	Refinement of microcracks enhanced the strengths.
[57]	GO suspension purchased, and age of GO unknown.	0.05% (compression) 0.01% (tension) 0.1% (flexural)	-	28-day increased by 32%.	(Direct method) 28-day increased by 26%.	28-day increased by 20%.	Enhancement of strengths occurred as GO sheets bridged via covalent bonding with CSH, improving its uniformity. In addition, propagation of cracks are deferred by delaying the formation of micro-cracks.
[58]	GO suspension purchased, and age of GO unknown.	-	-	-	-	-	GO nanosheets act as 2D platforms promoting the growth of CSH by means of the Stranski–Krastanov growth mode. Leading to a densified cement matrix.
[59]	Modified Hummers method, age of GO unknown.	0.05%	-	28-day increased by 32.71%.	(Direct method) 28-day increased by 18.93%.	-	Addition of GO in cement composites promotes hydration, reduces pore volume, accelerates crystallite formation, and improves alignment of crystallites.
[60]	GO suspension purchased, and age of GO unknown.	0.03% (compression) 0.05% (flexural)	-	28-day increased by 12.4%.	-	28-day increased by 16.10%.	GO promotes hydration, provides a filling effect and pattern role in the matrix. Moreover, the nano-filling effect eliminates majority of the pores in the mortar and rises the density.

**Table 2:** Weight of GO in different cement mortar samples used for testing.

Sample Name	Percentage of GO w.r.t to Cement (%)	Dry GO Required * (g)	GO Suspension Required ^#^ (g)
0 LGO	0	0	0
0.02 LGO	0.02	0.64	86.72
0.025 LGO	0.025	0.8	108.40
0.03 LGO	0.03	0.96	130.08
0.035 LGO	0.035	1.12	151.76
0.04 LGO	0.04	1.28	173.44

* Dry GO required was calculated w.r.t 3200 g of cement per batch mix ^#^ GO suspension with 0.0738% dry GO concentration.

## Data Availability

The generated data during the study are available from the corresponding author upon request.

## Supplementary Materials

The following supporting information can be downloaded at: https://www.mdpi.com/article/10.3390/nano13010018/s1, Table S1: Atomic percentages of elements at different areas of Figure 8e–h.
