# Supplementary material for: Influence of Laboratory Synthesized Graphene Oxide on the Morphology and Properties of Cement Mortar
Source: Nanomaterials (Basel). 2022 Dec 21;13(1):18. doi: 10.3390/nano13010018 (PMC9824886; doi:10.3390/nano13010018)
Supplement: Supplementary file 1 [file nanomaterials-13-00018-s001.zip › nanomaterials-2061539-supplementary.pdf]

Supplementary Table S1. Atomic percentages of elements at different areas of Figures 8 (e-h).

Figures and Areas	Atomic percentages (%)									
	**C**	**O**	**Na**	**Mg**	**Al**	**Si**	**Mo**	**K**	**Ca**	**Fe**
8 (e), Area 1	6.61	65.64	-	0.36	1.54	5.66	0.27	-	19.12	0.80
8 (e), Area 2	0.04	51.84	-	-	1.76	4.78	0.70	0.42	38.24	2.22
8 (f), Area 1	-	67.81	-	-	0.45	30.64	-	-	1.10	-
8 (f), Area 2	6.08	63.35	-	-	1.34	4.69	0.75	-	23.80	-
8 (f), Area 3	5.05	59.91	-	-	0.73	4.52	-	-	29.79	-
8 (g), Area 1	-	27.70	0.85	-	6.65	31.71	-	4.35	25.29	3.45
8 (g), Area 2	-	47.63	0.56	-	3.22	13.65	-	2.23	30.47	2.15
8 (g), Area 3	-	63.74	0.85	-	1.90	4.34	-	0.90	27.43	0.84
8 (h), Area 1	0.05	40.73	-	-	0.96	9.76	0.38	0.72	46.42	0.98
8 (h), Area 2	0.03	62.95	-	0.47	1.40	7.55	-	0.48	26.40	0.72
